# Structure and Functional Characteristics of Novel Polyurethane/Ferrite Nanocomposites with Antioxidant Properties and Improved Biocompatibility for Vascular Graft Development

**DOI:** 10.3390/polym17020152

**Published:** 2025-01-09

**Authors:** Marija V. Pergal, Jelena Brkljačić, Dana Vasiljević-Radović, Miloš Steinhart, Sanja Ostojić, Biljana Dojčinović, Bratislav Antić, Gordana Tovilović-Kovačević

**Affiliations:** 1 Institute of Chemistry, Technology and Metallurgy—National Institute of the Republic of Serbia, University of Belgrade, Njegoševa 12, 11000 Belgrade, Serbia; dana.vasiljevic@ihtm.bg.ac.rs (D.V.-R.); bmatic@chem.bg.ac.rs (B.D.); 2 Department of Biochemistry, Institute for Biological Research “Siniša Stanković”—National Institute of the Republic of Serbia, University of Belgrade, Bulevar despota Stefana 142, 11060 Belgrade, Serbia; brkljacic@ibiss.bg.ac.rs; 3 Institute of Macromolecular Chemistry CAS (IMC), Heyrovsky Sq. 2, 16206 Prague 6, Czech Republic; stein@buphy.bu.edu; 4 Institute of General and Physical Chemistry, University of Belgrade, Studentski trg 12-16, 11000 Belgrade, Serbia; ostojicsanja404@gmail.com; 5 The VINČA Institute of Nuclear Sciences—National Institute of the Republic of Serbia, University of Belgrade, Mike Petrovića Alasa 12-14, 11001 Belgrade, Serbia; bantic@vin.bg.ac.rs

**Keywords:** polyurethane hybrid films, nanoferrites, structural properties, nanomechanical properties, biocompatible polymers, vascular graft

## Abstract

Novel ferrite/polyurethane nanocomposites were synthesized using the in situ polymerization method after the addition of different spinel nanoferrite particles (copper, zinc, and copper–zinc) and examined as potential coatings for medical devices and implants in vascular tissue engineering. The influence of the nanoferrite type on the structure and functional characteristics of the polyurethane composites was investigated by FTIR, SWAXS, AFM, TGA, DSC, nanoindentation, swelling behavior, water contact angle, and water absorption measurements. Biocompatibility was evaluated by examining the cytotoxicity and adhesion of human endothelial cells and fibroblasts onto prepared composites and performing a protein adsorption test. The antioxidant activity was detected by UV–VIS spectroscopy using a 1,1-diphenyl-2-picrylhydrazyl (DPPH) scavenging assay. Embedding the different types of nanoparticles in the polyurethane matrix increased phase mixing, swelling ability, and DPPH scavenging, decreased surface roughness, and differently affected the stiffness of the prepared materials. The composite with zinc ferrite showed improved mechanical properties, hydrophilicity, cell adhesion, and antioxidant activity with similar thermal stability, but lower surface roughness and crosslinking density compared to the pristine polyurethane matrix. The in vitro biocompatibility evaluation demonstrates that all nanocomposites are non-toxic, exhibit good hemocompatibility, and promote cell adhesion, and recommends their use as biocompatible materials for the development of coatings for vascular implants.

## 1. Introduction

Polyurethanes (PUs) are polymers with great versatility due to the range of chemistries that can be employed in their synthesis, which results in a variety of structures and properties [1]. Poly(dimethylsiloxane) (PDMS)-based PUs have excellent mechanical, physical, and chemical properties [2,3]. The microphase-separated structure of PU allows it to be adapted to a large number of practical applications, i.e., thermal protective coatings, elastomers, gas separation membranes, industrial parts, construction materials, footwear, and sportswear [4,5,6]. In addition, PDMS-based PUs have gained importance in biomedical applications and tissue engineering (bone implants, catheters, and scaffolds) due to their exceptional biocompatibility and higher thromboresistance compared to most other polymers [3,7,8]. Moreover, recent research has shown that PUs can exhibit shape memory behavior [9], electrical conductivity [10,11], and magnetic or dielectric properties [12], resulting in an even wider range of potential applications. PUs processed using PDMS prepolymers, diisocyanates, and hyperbranched polyesters (HBPs) are thermoset polymers. The introduction of HBPs as crosslinking agents in the synthesis of PUs significantly expands their application potential [13,14,15,16,17,18]. The high functionality of HBPs as versatile polymers that contain a large number of free end groups enables speedy curing and the formation of highly crosslinked PU structures that can be employed in the development of various coatings and adhesives. In addition, several advantages, such as chemical stability, remarkable mechanical properties, blood compatibility, and, above all, the low cost of these materials, favor the use of PU networks over linear PUs.

Recently, a special focus has been placed on the outstanding properties of hybrid materials [19,20,21,22]. PU-based nanocomposites (NCs) have attracted great research interest mainly because of their advanced thermal, surface, and mechanical properties and the fact that very strong polymer–matrix hydrogen bonding can occur [20,21,22]. Nanofillers generally confer very efficient mechanical reinforcement to NCs as they have a very high specific surface area [23]. Furthermore, nanofillers are also useful to impart specific optical [24], chemical [25], electrical [26], magnetic [27], or gas barrier properties [28] to the matrix. Examples of promising PU NCs are PUs filled with graphene [26], carbon nanotubes [29], MXene [30], clay [20], Fe_3_O_4_ [27] or nanosilica [21].

In addition, polymeric NCs applied in the form of polymer coatings represent one of the optimal solutions for improving the biological performance of various medical implants [31]. Ferrite nanoparticles are among the nanofillers that are considered particularly useful in the field of medical coatings. As iron oxide-based nanoparticles, ferrites exhibit several biocompatible properties, such as magnetic moment, high colloidal stability, chemical stability, and simple surface functionality available for the attachment of biological molecules [32]. In addition to their excellent biocompatible and non-toxic characteristics, these nanoparticles can have useful catalytic properties and show good electromagnetic attributes and low eddy current losses. Therefore, they are used as catalyzers and sensors in magnetic resonance imaging, as well as in anti-cancer and anti-bacterial therapy [27]. The incorporation of ferrite nanoparticles into different polymeric matrices is an emerging research area. Studies show that the mechanical, electrical, thermal, magnetic, and shape memory properties of PU in which ferrite nanoparticles are embedded are improved over pure PUs [32,33,34,35,36]. Shahrousvand et al. [27] prepared polyester-based PU/Fe_2_O_3_ NCs as tissue-engineered scaffolds. The addition of iron oxide-based nanoparticles increased the mechanical strength of the obtained PU NCs and provided them with suitable flexibility. In fact, adding nanoparticles to the polymer matrix improved the hydrophilicity and increased the surface roughness, cell attachment, tensile strength, and modulus of the PU/Fe_3_O_4_ NCs. These improvements are a consequence of strong interfacial adhesion between PU surfaces and the nanoparticles and the reinforcing effect the stiff nanoparticles provide to the material matrix. Ashjari et al. [33] prepared PU-NC by mixing magnetite nanoparticles and commercially available PU and reported significant enhancements in the mechanical and thermal characteristics of the NCs. In addition, recent studies also show the potential of CoFe_2_O_4_-embedded thermoplastic PU and polyvinylidene fluoride nanocomposites as flexible triboelectric sensors for healthcare and electromagnetic interference shielding materials [37,38]. As research in the field of ferrite-based polymer composites is still in its infancy, the diverse performances of these materials need to be further explored. Although the improvement of various properties of Fe_3_O_4_-based linear PU NCs has been reported in the literature [33], there is no report on thermoset PU/ferrite (copper or zinc) in the biomedical domain.

In the current study, novel NCs based on a thermoset polyurethane polymer as a matrix and three types of spinel ferrite nanoparticles (copper, zinc, and copper–zinc) as nanofillers were prepared (PU/ferrite nanocomposites, PUNFs) and characterized for biomedical applications. This is a starting point in the process of developing biomaterials that could be considered custom-tailored composites for the production of suitable coatings for medical devices and implants. Therefore, pristine and NC PU films were produced; the PUNFs had a small weight percentage (1 wt.%) of ferrite nanoparticles in the PU matrix. The in situ polymerization method was used to produce thin NC films with 60 wt.% of soft segment, and the structural, thermal, mechanical, and surface properties of the new PUNFs were analyzed by Fourier transform infrared (FTIR), small-angle and near wide-angle X-ray scattering (SWAXS), differential scanning calorimetry (DSC), thermogravimetric analysis (TGA), nanoindentation, atomic force microscopy (AFM), swelling behavior, water absorption, and water contact angle. In addition, biocompatibility was tested in vitro by LDH, MTT assay, and cell adhesion testing using endothelial and fibroblast cell lines, and following the adsorption of plasma proteins. The antioxidant properties of the novel PU-embedded nanoferrites were investigated using a 1,1-diphenyl-2-picrylhydrazyl (DPPH) radical scavenging assay.

## 2. Materials and Methods

### 2.1. Materials

Boltorn^®^ BH-20, a second pseudo-generation hydroxy-functional aliphatic hyperbranched polyester (*M*_n_ = 1350 g/mol, functionality = 12), was procured from Perstorp Specialty Chemicals AB (Perstorp, Sweden) [39] and dried at 50 °C for 5 h under vacuum before use. 4,4′-Methylene diphenyl diisocyanate (MDI, ≥98%) was obtained from Sigma-Aldrich. Poly(dimethylsiloxane) (PDMS) macrodiol (*M*_n_ = 1200 g/mol, determined by GPC) was supplied by Gelest (Frankfurt, Germany). PDMS is a linear, telechelic oligomer with terminal hydroxyl groups and contains one ethylene oxide unit. *N*-methyl-2-pyrrolidone (NMP, 99%, Acros) and tetrahydrofuran (THF, 98%, J. T. Baker) were dried by distillation over calcium hydride and lithium aluminum hydride, respectively. The reaction catalyst was stannous(II)-ethylhexanoate (Sn(Oct)_2_, Sigma-Aldrich, St. Louis, MO, USA).

For the synthesis of nanoferrites (MFe_2_O_4_; M = Zn, Cu, Cu_0.5_Zn_0.5_), iron(III) chloride hexahydrate (FeCl_3_·6H_2_O, ≥98%, reagent grade), zinc chloride (ZnCl_2_, ≥98%, reagent grade), copper(II) sulfate pentahydrate (CuSO_4_·5H_2_O, ≥98.0%, ACS reagent), and ammonium hydroxide solution (NH_4_OH, 28.0–30.0% NH_3_ basis, ACS reagent) were purchased from Sigma Aldrich.

### 2.2. Preparation Procedure

#### 2.2.1. Ferrite Nanoparticle Preparation and ICP-OES Analysis

The synthesis was carried out in an inert nitrogen atmosphere. Appropriate amounts of CuSO_4_∙5H_2_O, ZnCl_2_, and FeCl_3_·6H_2_O salts were dissolved in 320 mL of deionized water. The total amount of salt mixture used in the synthesis corresponded to 0.030 mol Fe^3+^ and (1) 0.015 mol Cu^2+^, or (2) 0.015 mol Zn^2+^, or (3) 0.0075 mol Cu^2+^ and 0.0075 mol Zn^2+^ for CuFe_2_O_4_, ZnFe_2_O_4_, and Cu_0.5_Zn_0.5_Fe_2_O_4_, respectively. After the salts were completely dissolved, 22.5 mL of ammonium hydroxide (ACS reagent, 28.0–30.0% NH_3_ basis, Sigma Aldrich) was slowly added at a constant rate over a period of 60 min while continuously stirring. The final pH of the mixture was 10.

In the next step of the synthesis, the resulting precipitate was treated with microwave-assisted hydrothermal treatment. The total volume of the mixture with the coprecipitate (350 mL) was divided into seven Teflon vessels, each with a volume of 100 mL. A 50 mL portion of the mixture was added to each vessel, which is the maximum allowed volume per vessel. The vessels were placed in a segmented rotor (HPR-1000/10S high pressure) and heated in a microwave digestion system (ETHOS 1, Advanced Microwave Digestion System, MILESTONE, Sorisole, Italy). The temperature program was set so that the microwave power ranged from 0 to 1000 W, with linear heating of the mixture from room temperature to 150 °C over 10 min. Once the mixture reached 150 °C, the temperature was maintained at a constant for an additional 20 min at 150 °C. The maximum pressure in the reaction vessels was declared at 100 bar.

After completing the microwave-assisted hydrothermal synthesis, the vessels were rapidly cooled in an air stream, and the contents from all the vessels were combined in one beaker. The resulting precipitate was washed with demineralized water, with decantation assisted by an external magnet. The washing process continued until a negative test for chlorides and sulfates was achieved, which was verified by performing classical qualitative tests for these anions. Conductivity measurements of the supernatant (the conductometric method) further confirmed the complete washing. The precipitate was then dispersed in 50 mL of water and dried in an oven at 60 °C. After drying, the precipitate was ground in an agate mortar. The samples were poorly crystalline or amorphous, so they were calcined at 300 °C for 2 h to ensure complete crystallization.

A volume of 250 µL of the nanomaterial suspension was measured using a micropipette and dissolved in 5 mL of concentrated nitric acid (HNO_3_, p.a. 65% m/m, Sigma Aldrich) with heating. The resulting solution was diluted to a total volume of 250 mL in a standard volumetric flask. The concentrations of Cu, Zn, and Fe in the prepared solution were measured using the analytical technique of inductively coupled plasma optical emission spectrometry (ICP-OES) with an iCAP 6500 Duo ICP instrument (Thermo Fisher Scientific, Cambridge, UK), and certified standards from Alfa Aesar GmbH & Co KG (Karlsruhe, Germany) were used to prepare calibration solutions within the required concentration range. Measurement reliability was confirmed by the relative standard deviation (RSD), which was less than 0.5%. The quality of the analytical process was validated using a certified reference material (CRM), EPA Method 200.7 LPC Solution, provided by ULTRA Scientific, USA. The recovery of the measured metal concentrations compared to the certified value was 99.7–99.9%.

#### 2.2.2. Polyurethane/Ferrite Nanocomposite Material Preparation

**Scheme 1:**
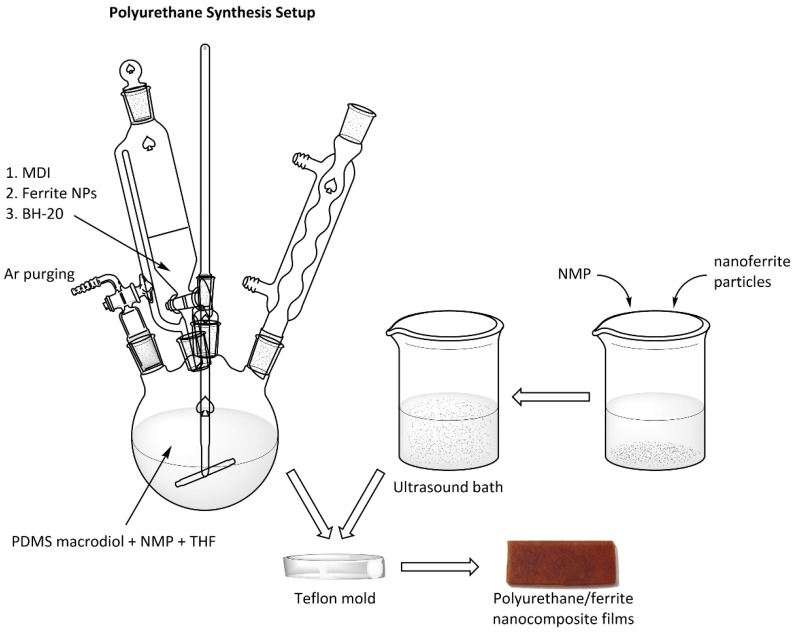
Schematic representation of the synthesis procedure of polyurethane/ferrite nanocomposite films.

The PU/ferrite nanocomposites were prepared in situ using a two-step polymerization procedure (Scheme 1). A series of PUNFs containing 60 wt.% of a soft PDMS segment with copper, zinc, and copper–zinc ferrite nanoparticles were prepared. Each sample contained 1 wt.% of ferrite nanoparticles, calculated based on the dry weights of the nanofiller and PU. The [NCO]/[OH] total molar ratio was set at 1.05 (where [OH]_total_ = [OH]_PDMS_ + [OH]_BH-20_) [21].

The PDMS macrodiol, MDI, catalyst (0.15 mol.% Sn(Oct)_2_ per mol PDMS macrodiol), and NMP/THF (9/1 *v*/*v*) were mixed in a round-bottomed flask at 60 °C for 45 min at 900 rpm. After this, ferrite nanoparticles dispersed in the NMP (using an ultrasonicator at 25 °C for 20 min) were added to the reaction mixture, and it was constantly stirred for 15 min at 60 °C. This first phase of polyaddition was monitored by the dibutylamine back-titration method [40]. In the second phase, a dilute solution of BH-20 in the NMP (20 wt.%) was added to the NCO-terminated prepolymer and mixed continuously at 70 °C for 1 h. Each mixture was then poured onto Teflon plates to obtain a dry film thickness of ~500 μm. The films were dried in a force-draft oven at 50 °C for 3 h, at 80 °C for 24 h, and then under vacuum at 60 °C for 5 h, resulting in brown, continuous films. A pristine PU film (0 wt.% nanofiller) was prepared under the same conditions. The naming codes (e.g., PUNF-60-Cu) indicate the wt.% of the soft segment and the type of ferrite used (e.g., 60 wt.% soft segment and copper ferrite in PUNF-60-Cu).

### 2.3. Methods of Characterization

The FTIR spectra of the polymer films were recorded in ATR mode using a Nicolet Magna System 560 spectrophotometer, with a scanning range of 400 to 4000 cm^−1^, a resolution of 4 cm^−1^, and 64 scans per sample. Peak separation of the -NH and C=O bands in PUNFs was performed by peak deconvolution (using Peak.Fit software v4.12).

Materials were characterized by small- and wide-angle X-ray scattering (SWAXS) using a two-pinhole point-focusing SAXS instrument (originally Molecular Metrology, Northampton, MA, USA, upgraded by SAXSLAB, now Xenocs). A CuKα beam (λ = 0.154 nm) generated by Rigaku Micromax-003 was detected by a 2D Pilatus3 300 K detector at distances of 0.055, 0.401, and 1.402 m from the sample. Data were azimuth averaged and adjusted to absolute scale using a glassy carbon standard, covering a q interval from 0.05 to 35 nm^−1^. After merging the data from these positions, the reliable q interval from 0.05 to 35 nm^−1^ was reached, where q is the magnitude of the scattering vector q = 4π sin(θ)/λ, and θ is half of the scattering angle. The highest scattering angle reached corresponds to 50° 2θ.

SEM images of the fractured surfaces were obtained with a JEOL JSM-6610LV at 20 kV and a working distance of ~10 mm. Samples were cryo-fractured in liquid nitrogen and gold-coated before analysis.

Nanoindentation measurements were performed using an Agilent G200 instrument. The applied load was 30 nN, with the depth control mode set at 45 μm and Poisson’s ratio set to 0.49. For each sample, 100 measurements were taken in a 10 × 10 array.

Thermogravimetric analysis (TG) was performed using a TA Instruments TGA Q500 under an inert N_2_ atmosphere (flow rate 60 mL/min) from room temperature to 700 °C at a heating rate of 5 °C/min. Film samples weighed ~4–5 mg.

Differential scanning calorimetry (DSC) experiments were conducted using a DSC Q1000 (TA Instruments, New Castle, DE, USA) with an RCS cooling unit, as previously described [20]. Samples (~5 mg) were placed in aluminum crucibles and scanned from −90 to 200 °C at heating and cooling rates of 10 and 5 °C/min, respectively, under an N_2_ purge flow of 50 mL/min.

Water absorption and swelling behavior were investigated by immersing the materials in distilled water and THF for 72 h at room temperature, as previously described [22]. Rectangular test samples (10.0 mm × 20.0 mm × 1.0 mm ± 0.2 mm) were immersed in the medium, and their weight was measured at regular intervals until equilibrium swelling was reached. The swelling parameters in THF (the values of the swelling degree; the volume fraction of the crosslinked polymer in the swollen specimen, V_PUNF_; the crosslinking density, ν; and the number average molecular weight of polymer chain between crosslinks, M_c_) of prepared PUNFs were calculated [20,22].

The contact angles were measured using an optical goniometer with a digital camera, following Zisman’s method [41]. The results are mean values of three replicates.

The surface topography was investigated by atomic force microscopy (AFM) using an NTEGRA Prima microscope (NT-MDT, Moscow, Russia) under ambient conditions. Silicon n-type, antimony-doped probes with Au coating were used. Image analysis was performed with the WSxM 4.0 software version Beta 9.3 [42], with data expressed in height and phase modes at a 2 × 2 μm^2^ scan size.

#### Extraction and Metal Ion Analysis

The PUNF films were extracted according to Pergal et al. (2022) [22], with the exception that we used rectangular films (10.0 mm × 15.0 mm × 1.0 mm ± 0.2 mm). Concentrations of metal ions were analyzed using MP-AES (MPAES 4200, Agilent Technologies, Santa Clara, CA, USA), as reported previously [22].

### 2.4. Biocompatibility Characterization

#### 2.4.1. Protein Adsorption Determination

The protein adsorption capacity of PUNFs was investigated under the same conditions as previously reported by us for other materials [43]. Samples were incubated in a mixture of proteins dissolved in phosphate-buffered saline (PBS, 8.7 g L^−1^ K_2_HPO_4_, 6.8 g L^−1^ KH_2_PO_4_, and 8.76 g L^−1^ NaCl, pH 7.4) for 2 h at 37 °C. The adsorbed proteins were separated electrophoretically on 6% SDS-polyacrylamide gels under non-reducing conditions. After staining the gels with Coomassie Brilliant Blue R-250, the protein bands were visualized by iBright and quantitatively analyzed using iBright Analysis software (v5.1.0.). The amount of adsorbed proteins was calculated using standard curves generated simultaneously with the samples. The results are expressed as µg protein/cm^2^ film.

#### 2.4.2. Cell Lines

The human endothelial cell line EA.hy926 and human fibroblast MRC-5 cells were obtained from the American Type Culture Collection (ATCC, Manassas, VI, USA). The EA.hy926 cells were incubated in high glucose Dulbecco’s Modified Eagle Media supplemented with 10% fetal bovine serum, 2 mM L-glutamine, 100 mg/cm^3^ streptomycin, 100 U/cm^3^ penicillin, and 20% HAT media supplement (all from Gibco, Thermo Fisher Scientific, Waltham, MA, USA). Human fibroblast MRC-5 cells were cultivated in RPMI medium supplemented with 10% fetal bovine serum and 1% antibiotic/antimycotic solution (all from Gibco, Thermo Fisher Scientific, Waltham, MA, USA). The cultures were maintained in a humidified atmosphere with 5% CO_2_ at 37 °C and prepared for the experiments using the conventional trypsinization procedure.

#### 2.4.3. Cell Viability and Cytotoxicity Assessment

The activity of mitochondrial dehydrogenase, as a measure of cell viability and cell membrane integrity, was examined using MTT (3-[4,5-dimethylthiazol-2-yl]-2,5-diphenyltetrazolium bromide) and LDH (lactate dehydrogenase) assays, respectively. EA.hy926 cells were seeded on sterilized PU films (20,000 cells per well), and viability was assessed 48 h post-seeding. In brief, after incubation with MTT solution (0.5 mg/mL) for 1 h, it was removed, and 100 µL dimethyl sulfoxide (DMSO) was added to each well. The formazan salts formed on the membranes were rinsed off with DMSO, the samples were removed from the wells, and the absorbance was measured spectrophotometrically at 570 nm (Multiskan spectrum, Thermo Scientific, Waltham, MA, USA). Mitochondrial activity was expressed relative to the control sample (cells grown with PU-60 sample films), taken as 100%. LDH activity was determined by mixing 50 µL of the cell culture medium with 50 µL of the LDH substrate (lactic acid 54 mM, phenazine methosulfate 0.28 mM, p-iodonitrotetrazolium chloride 0.66 mM, and β-NAD 1.3 mM) and measuring the absorbance at 492 nm (Multiskan spectrum, Thermo Scientific). LDH activity, as a cytotoxicity index, was calculated relative to the control (cells grown without added material and lysed with Triton X-100), taken as 100%, as previously described [43].

#### 2.4.4. Cell Adhesion

The adhesion of endothelial cells and fibroblasts on the surface of the PU films was investigated by light microscopy using a computer-controlled Carl Zeiss Axiovision microscope. The cells were seeded on PU films placed in 96-well plates (Sarstedt, Nümbrecht, Germany) at a density of 10,000 cells/well. The cell attachment was assessed 96 h after seeding. The attached cells were fixed with paraformaldehyde solution (4%), stained with nigrosine solution (0.4%), and visualized with the Carl Zeiss Axiovision microscope [43]. The possible influence of surface preconditioning with the three major plasma proteins bovine serum albumin (BSA), bovine γ-globulin (BGG), and fibrinogen (FBG) on cell adhesion was investigated after pretreatment of the PU films with the three-component mixture of BSA (40 mg/cm^3^), BGG (10 mg/cm^3^), and FBG (3 mg/cm^3^). The samples were incubated in the protein mixture at 37 °C for 2 h before cell seeding, and the attached cells were photographed 96 h after seeding, as described previously.

#### 2.4.5. Antioxidant Activity Assay

The antioxidant properties of PU-embedded nanoferrites were determined using a 1,1-diphenyl-2-picrylhydrazyl (DPPH) scavenging assay. The test was performed as described in Baheiraei et al. [11], with minor modifications. The materials were immersed in a methanol solution of DPPH (100 µM) and incubated in the dark for 30 min. After incubation, the absorbance of the DPPH solution at 517 nm was determined spectrophotometrically (BioTek Synergy H1 multimode reader, Agilent, Santa Clara, CA, USA), and the percentage of DPPH degradation was calculated using the following formula:

% of DPPH scavenging = (A_DPPH_ − Asample)/A_DPPH_ × 100

A_DPPH_ = absorbance of DPPH solution without membranes

Asample = absorbance of DPPH solution incubated with membranes

In addition, the UV–VIS spectra of the solution (range 450–580 nm) were recorded with a spectrophotometer (BioTek Synergy H1 multimode reader, Agilent, USA).

### 2.5. Statistical Analysis

Data were statistically analyzed using one-way ANOVA, followed by post-comparison Bonferroni’s test. * indicates *p* < 0.05; ** *p* < 0.01; and *** *p* < 0.001.

## 3. Results and Discussion

**Scheme 2:**
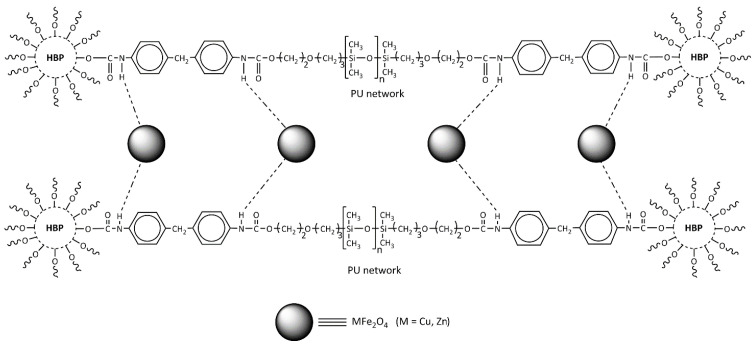
Basic chemical structure of the prepared polyurethane/ferrite nanocomposites.

The novel PU nanocomposites investigated here were synthesized from PDMS macrodiol (soft segment), Boltorn^®^ (Perstorp Specialty Chemicals AB, Perstorp, Sweden) hyperbranched polyester of the second pseudo-generation (BH-20), and MDI (parts of the hard segments) in the presence of different types of ferrite nanoparticles (copper, zinc, and copper–zinc) (1 wt.% in the polymer matrix) (Scheme 2). The content of the soft segment was 60 wt.% in all prepared PU NCs.

The chemical composition of the synthesized ferrite nanoparticles was analyzed using ICP-OES. Based on the concentrations of Cu, Zn, and Fe, converted to molar ratios, the stoichiometric formulas of the synthesized nanoferrites were determined as follows: Cu_0.938_Fe_2.062_O_4_, Zn_0.655_Fe_2.345_O_4_, and Cu_0.248_Zn_0.372_Fe_2.380_O_4_. These results indicate partial deviations of the stoichiometric formulas from the nominal ones: CuFe_2_O_4_, ZnFe_2_O_4_, and Cu_0.5_Zn_0.5_Fe_2_O_4_. The greatest deviation was observed in the incorporation of zinc into the spinel structure of ferrites. For clarity, the samples are referred to by their nominal compositions in the following text.

The TEM image of the synthesized nanoparticles is shown in Figure S1 (Supplementary Materials). It can be seen that the ferrite nanoparticles show a semispherical shape and have a small size on the nanoscale. As can be seen from the TEM images, nanoparticles can aggregate due to their high surface energies and magnetic attractive forces [44]. The estimated average particle sizes were ~5, ~3, and ~9 nm for CuFe_2_O_4_, ZnFe_2_O_4_, and Cu_0.5_Zn_0.5_Fe_2_O_4_, respectively.

The crystal symmetry of the synthesized samples was examined using X-ray diffraction data obtained with a SmartLab^®^ X-ray diffractometer (Rigaku, Tokyo, Japan), utilizing Cu Kα radiation (λ = 0.1542 nm). Analysis of the diffraction patterns (Figure S2 (Supplementary Materials)) confirms the expected crystal structure. All samples crystallize in a spinel-type structure with the space group Fd3m. Weak reflections corresponding to the CuO phase were observed in the CuFe_2_O_4_ sample.

This study is the first to investigate the properties of nanoferrite-loaded PU obtained using in situ polymerization for potential use in biomedical applications, i.e., coatings for medical devices and implants. A key step in PU NC production is an excellent, homogeneous dispersion of the nanoparticles in the PU matrix, as previously described [35,45]. The main advantage of in situ polymerization is that the nanoparticles are better mixed into the pre-polymer compared to pre-formed polymers produced by other preparation methods, such as melt mixing or solution blending methods [33,35].

### 3.1. FTIR Analysis

**Figure 1:**
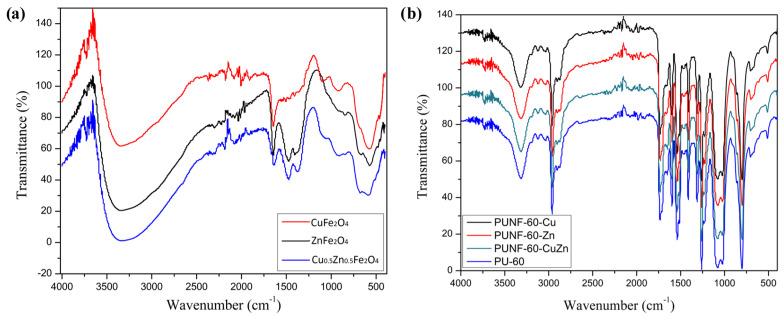
FTIR spectra of the prepared (**a**) ferrite nanoparticles and (**b**) polyurethane/ferrite nanocomposites and pure PU films.

FTIR analysis was used to depict the IR spectra of the ferrite nanoparticles, pristine PU, and PUNFs as presented in Figure 1. In all spectra of the ferrite nanoparticles, the strong peak at around 580 cm^−1^ and the small peak at around 490 cm^−1^ were assigned to the vibrational frequency of Fe-O and the stretching vibration of the M-O band (M= Cu, Zn, Cu_0.5_Zn_0.5_) [46]. In addition, the O–H stretching vibration of H_2_O could be associated with the peaks observed at around 3350 cm^−1^ due to moisture absorption in the ferrite nanoparticles [46,47].

The typical bands related to the polyurethane structure were observed in the FTIR spectra of pristine PU and PUNFs (Table 1) [46]. The complete consumption of isocyanate groups is proved by the absence of NCO stretching vibrations at around 2270 cm^−1^ in all materials.

Having in mind that the -NH groups in the urethane linkage can form hydrogen bonds with urethane carbonyl, ester carbonyl, and ether oxygen in poly(urethane-siloxane), the -NH and carbonyl peaks were separated to examine this more closely. Stretching bands near 3324 cm^−1^ (strong peak) and 3450 cm^−1^ (weak peak) were observed, which correlate to the hydrogen-bonded and free (non-hydrogen-bonded) NH stretching vibrations, respectively. This suggests that almost all amide groups in PUNFs are engaged in hydrogen bonding.

The deconvolution data for the carbonyl and -NH peaks are listed in Table 2. The hydrogen bonding index (HBI) of the urethane groups and the degree of phase separation (DPS) between soft and hard segments in the PU matrix [21,48,49,50] are shown (Table 2). Both the HBI and DPS of PUNFs were lower than those of the pristine PU network (Table 2). The low value of the HBI for the PUNFs suggests that the addition of ferrite nanoparticles decreases the amount of hydrogen-bonded carbonyl of the urethane groups in the ordered domains. Therefore, it was found that the ferrite nanoparticles strongly modify the morphology of PU by reducing the phase separation between the hard and soft segments, i.e., by increasing the phase mixing. The material PUNF-60-Zn showed the highest values for the HBI and DPS in the series of prepared PUNFs. It was found that the addition of zinc ferrite to the PU matrix did not cause a significant decrease in the HBI and DPS compared to pure PU (Table 2). Furthermore, PUNF-60-Zn showed the highest values for the hydrogen-bonded urethane carbonyl groups and, at the same time, the lowest values for the free urethane carbonyl groups, confirming that most of the carbonyl groups were involved in hydrogen bonding and indicating better microphase separation compared to other prepared PUNFs. Additionally, the mild shifts (to lower wavelengths) in the PUNFs’ band positions of carbonyl urethane hydrogen bonding in the ordered domains and -NH urethane hydrogen-bonded band positions were attributed to the interruption of the hydrogen bonds between the polymer chains in the nanocomposites by the ferrite nanoparticles.

In the PUNFs, the percentage of -NH groups crosslinked with hydrogen bonds ranged from 85.8 to 94.0%, less than that of pristine PU (95.1%), confirming that ferrite nanoparticles hindered hydrogen bonding between -NH and carbonyls (shown in Table 2).

We also propose an interaction between the oxygen from CuFe_2_O_4_, ZnFe_2_O_4_, and Cu_0.5_Zn_0.5_Fe_2_O_4_ with the -NH in the urethane bonds, thus forming complexes, as was suggested earlier [35]. Furthermore, our work supports the fact that urethane’s carbonyl group can react with the minimal amount of –OH absorbed by the nanoparticles, as suggested by others [35].

It can be assumed that the ferrite nanoparticles insert into the polymer structure during in situ polymerization as proposed in our study and shown in Scheme 2, where it is suggested that the main interaction between the polymer and MFe_2_O_4_ is achieved by hydrogen bonding.

### 3.2. SWAXS Analysis

**Figure 2:**
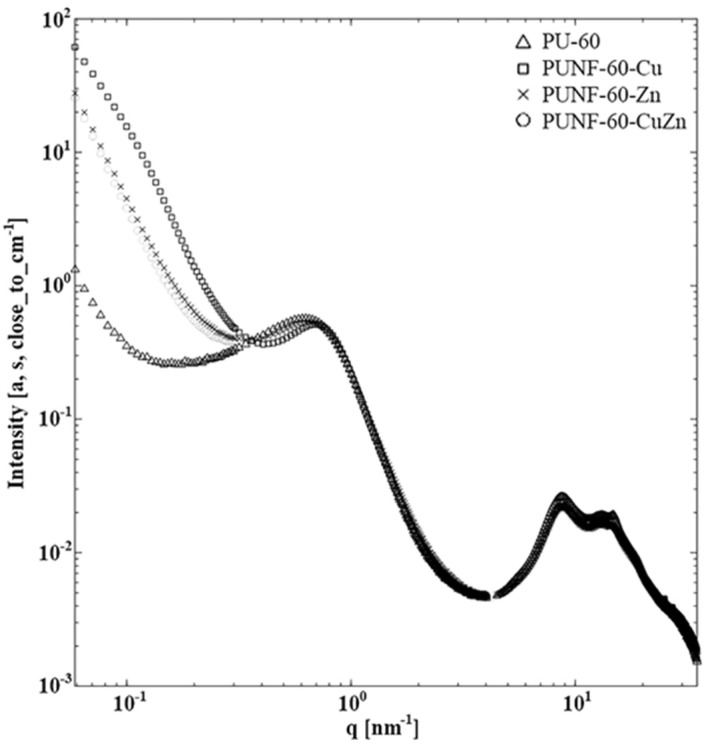
X-Ray scattering of the prepared PU/ferrite nanocomposites and pristine PU films in the whole small-angle and near wide-angle experimental region.

Figure 2, which covers the entire SWAXS experimental region, shows that the scattering curves were hardly distinguishable up to the first maximum, i.e., q = 1 nm^−1^. The differences were highlighted when the scattering of the pure PU-60 system was subtracted. At the lowest qs, it is evident that the system does not correspond to particles but rather to globular regions whose size was too large, so that only the tails of the SAXS scattering curves were visible; for this reason, we can only estimate some parameters and compare them between the materials. Table 3 shows the estimated values for the radius of gyration Rg [nm^−1^], the intensity at zero angle I0+, the slopes, the lamellar distance [nm], and the size of the domains from the first minimum R1m [nm]. It must be stressed that we are working with rough estimates, or rather pseudo-parameters, obtained under conditions far from those expected by SWAXS theory. However, our rough estimates are consistent with the interpretation in the literature. Most spectacular was a steep upward jump at I0+ and a downward jump at R1m. Both changes can be attributed to the use of heavy atoms as nanofillers. When using the position of the first minima, we must consider that the scattering when using nanofillers is the superposition of information from these and the original network. The lowest angular region of the PUNF-60-Cu curve was clearly different from the rest as it has two distinct slopes. This can probably be attributed to the higher roughness of the filler nanoparticles. This explanation is supported by the AFM results (see below) and the higher protein adsorption (see below), while the other parameters were similar to those of the other measured systems.

### 3.3. SEM Analysis

**Figure 3:**
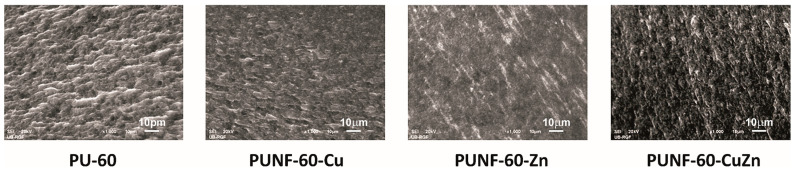
SEM micrographs of the cross-sectional surface of the prepared pure PU and polyurethane/ferrite nanocomposite materials.

SEM micrographs of the PUNFs and the pristine PU films are shown in Figure 3. The homogeneous dispersion of the ferrite nanoparticles in the polymer matrix was observed in these SEMs, showing the preparation method we used was suitable for this purpose. The PUNFs exhibited a lower surface roughness than pristine PU (Figure 3).

### 3.4. Nanoindentation Analysis

The nanomechanical properties (mechanical properties at the nanoscale) were evaluated and are shown in Table 4.

The resistance of a material to local surface deformation is indicated by the measured hardness, while its stiffness is characterized by the elastic (Young’s) modulus. In our study, the different types of ferrite nanoparticles affected the mechanical properties of PU NCs: both the elastic modulus and hardness were greater in PUNF-60-Zn (184 MPa and 41 MPa) and lower in PUNF-60-Cu (152 MPa and 31 MPa) and PUNF-60-CuZn (142 MPa and 27 MPa) compared to pristine PU (170 MPa and 31 MPa). The addition of zinc ferrite nanoparticles in PU increased Young’s modulus and hardness of PUNF-60-Zn by 8.23% and 32.25%, respectively, compared to pristine PU, meaning the nanocomposite was stiffer. This effect could be correlated with efficient reinforcement and the fact that the zinc ferrite nanoparticles were very well dispersed in the polymer matrix. Certainly, these results show the improved stiffness and hardness of the material with zinc ferrite nanoparticles incorporated into the PU matrix. The higher Young’s modulus (Table 4) for the ZnFe_2_O_4_ nanoparticle-based PUNF also shows better compatibility and dispersion of ZnFe_2_O_4_ than CuFe_2_O_4_ and Cu_0.5_Zn_0.5_Fe_2_O_4_ nanoparticles in the polymer matrix. The large surface area of the nanoparticles and the strong interaction between the nanoparticles within the PU matrix account for the improved elastic modulus of PUNF-60-Zn. Furthermore, the observed less desirable mechanical properties of the other NCs we prepared could be due to the poorer dispersion of the copper- and copper–zinc ferrite nanoparticles, which led to more defects in the composite networks.

The prepared PUNFs exhibited higher Young’s moduli (from 0.7 to 7.3 MPa) compared to the PDMS-based PU NCs reported in the literature [51], which is probably due to the exact type of ferrite nanoparticles and the dispersion and production methods we used to produce the PUNFs.

Additionally, the investigated PUNF materials were less plastic than the pristine PU (Table 3), reflecting the extent of plastic indentation [52,53] and fracture toughness of the new materials, as previously noted for other materials [52,54].

### 3.5. Thermal Characteristics

**Figure 4:**
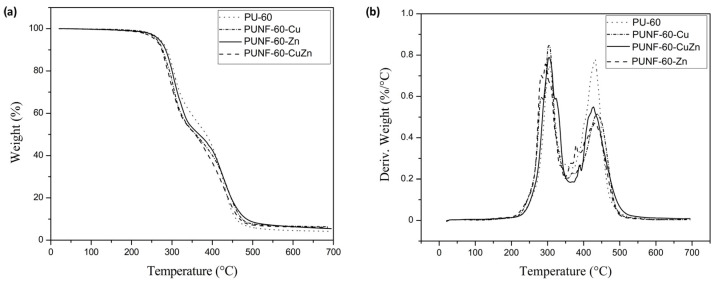
(**a**) TGA thermograms and (**b**) DTG curves for nanoparticles, pristine PU, and polyurethane/ferrite nanocomposites.

The TGA thermograms of the pristine PU and PU NC films in a nitrogen atmosphere are presented in Figure 4. T_10%_ denotes the temperature at which a material starts to degrade, relevant for its thermal stability (Table 5). The start degradation temperature decreased in the following order: PUNF-60-Zn > PUNF-60-CuZn > PUNF-60-Cu (Table 5). These data show that the T_10%_ for PUNF-60-Cu (T_10%_ = 283 °C) was slightly lower compared to the other prepared PUNFs (T_10%_ = 286 and 285 °C for PUNF-60-Zn and PUNF-60-CuZn) and pristine PU (T_10%_ = 287 °C). This can be attributed to phase mixing, which causes significant weight loss in the initial phase of thermal degradation. This observation suggests that nanoparticles, especially copper nanoferrites, disrupt the hydrogen bonds in the hard segments and, therefore, determine a lower temperature for chain degradation, which also increases the decomposition rate [36]. It is obvious that the temperature of the first decomposition peak was higher for the prepared PU NCs than for pristine PU. This indicates a higher thermal stability of the prepared PU NCs compared to pristine PU. Only PUNF-60-Zn showed comparable thermal stability (T_10%_ = 286 °C) compared to pure PU (T_10%_ = 287 °C). The prepared PUNF with zinc ferrite nanoparticles exhibited the best thermal stability compared to the other prepared PUNFs, which is probably due to the good compatibility and better dispersion of the ZnFe_2_O_4_ nanoparticles within the PU compared to CuFe_2_O_4_ and Cu_0.5_Zn_0.5_Fe_2_O_4_ nanoparticles.

According to the literature [35], it is worth mentioning that the thermal stability of PU-HBP/Fe_3_O_4_ NCs improved with the increasing nanoparticle content, as their homogeneous dispersion in the matrix imparted better absorption of the heat supplied to the system, and thus, these nanoparticles behaved like a heat insulator. Moreover, the thermal stability of the PUNFs prepared in this study was greater than that of the vegetable oil-based HBPU/Fe_3_O_4_-polyaniline NC [55].

Thermal decomposition of the pristine PU (Figure 4) started at 287 °C, and 4.33 wt.% ash remained at 600 °C (Table 5). Notably, the introduction of ferrite nanoparticles into the PU matrix had no effect on the thermal stability compared to pure PU. The greater residual weight percent at 650 °C for the PUNFs compared to pristine PU (Table 5) is likely due to the nanoferrites in the NCs.

The DTG curves for all materials showed four weight loss peaks. The decomposition of the hard segments in the PU matrix is related to the urethane bonds, which were thermally the weakest bonds, indicated by the first peak. The temperature of the first decomposition peak was higher for the prepared PU NCs than for pristine PU. The decomposition of the ester, ether, and PDMS components in the PU matrix is attributed to the second, third, and fourth peaks, respectively, and these occurred at successively higher temperatures (Figure 4b). The high thermal stability of PDMS was likely a consequence of the high bond strength of the Si-O bond, as we noted earlier [3].

**Figure 5:**
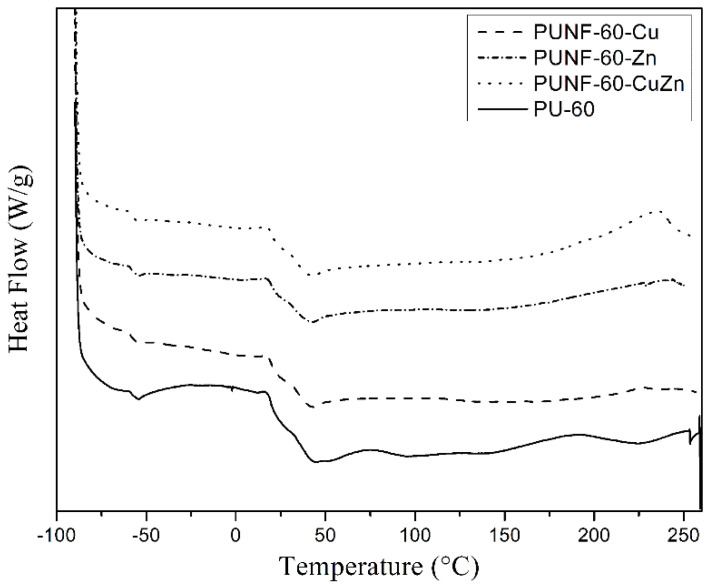
DSC curves of pristine PU and polyurethane/ferrite nanocomposites.

The DSC thermographs, measured in the range of −90 to 230 °C (Figure 5), show that each material had a glass transition temperature (T_gHS_) of hard segments. The T_gHS_ of the PUNFs ranged from 26 to 29 °C while that of pristine PU was 29 °C (Table 5). The T_gHS_ of the PUNF-60-Zn and PUNF-60-CuZn materials was lower than that of pristine PU. The relatively low T_gHS_ values indirectly indicate stronger interactions between the nanoparticles than between other components in the PUNFs. Thus, the free volume was increased, and the chain flexibility was enhanced by nanoparticle addition, as this disrupted the crosslinking of the polymer network [33,35]. The DSC thermograph results obtained agree with those of the crosslinking density of the prepared PUNFs (see below).

### 3.6. Swelling and Water Absorption Analysis

The swelling ability of the PUNFs was higher than that of pristine PU. The prepared PUNFs had lower values for V_PUNF_ and ν but higher M_c_ values than pristine PU. The prepared PUNFs had lower degrees of crosslinking density than the pristine PU (Table 6). In the series of PUNFs, PUNF-60-Zn had the lowest crosslinking density, while PUNF-60-CuZn had the highest.

Careful consideration of surface hydrophilicity and hydrophobicity is of great importance for the development of biomedical materials. In some applications, e.g., in blood vessels, the materials must have highly hydrophobic surfaces [43,44], whereas in tissue engineering applications and as coatings for medical devices, the materials should be moderately hydrophilic. The increase in mass over time was used as a measure of swelling behavior in water in the present study, and the results are shown in Table 6. The incorporation of ferrite nanoparticles into the PU matrix increased the amount of water absorbed (Table 6). This can be attributed to the inherent hydrophilicity of the ferrite nanoparticles incorporated into the PUNF materials. These results are in line with the water absorption of flexible magnetic PU/Fe_2_O_3_ NCs based on poly(ε-caprolactone) soft segments [27]. The PUNF-60-Zn material had the highest hydrophilicity in our series of prepared PUNFs, and this property may strongly influence its biocompatibility behavior.

### 3.7. Water Contact Angle Analysis

The interaction between solid and liquid phases is determined by surface wettability, and this interaction plays an important role in various biological systems and technological applications [56,57]. Static contact angle measurement was used to investigate the surface wettability of pristine PU and PU nanocomposites, and the results of the water contact angle (WCA) measurement are shown in Table 7. The WCA of PU NCs was lower for materials containing ferrite nanoparticles than for pristine PU. The WCA of pristine PU was 97°, which is considered relatively hydrophobic according to other studies [21,22]. The WCAs of PUNF-60-Cu and PUNF-60-CuZn show these materials had a relatively hydrophobic character compared to the PUNF-60-Zn material. Comparing the WCAs of our PU NCs, clearly the loading of smaller nanoparticles (ZnFe_2_O_4_ with an average size of 3 nm) led to a greater decrease in WCA than the loading of larger nanoparticles (CuFe_2_O_4_ with 5 nm and Cu_0.5_Zn_0.5_Fe_2_O_4_ with 9 nm) in the PU matrix (Table 7).

### 3.8. AFM Analysis

**Figure 6:**
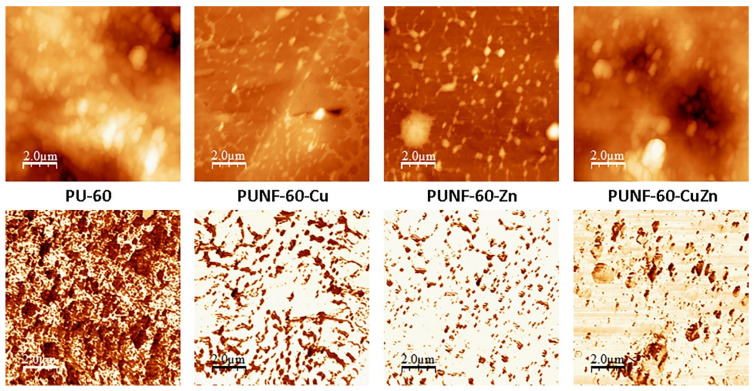
Two-dimensional AFM (**above**) and phase AFM (**below**) images of the prepared PUNFs and pure PU.

Surface morphology was visualized and measured using AFM images, while phase images showed the contrast between the hard and soft domains in the PU network, which is a measure of heterogeneity (Figure 6). The lighter (harder) and darker (softer) areas seen in the phase images originated mainly from the micro-phase separation of the soft and hard segments in the PUNFs. In all prepared materials, clusters of soft- and hard-segment-rich regions were observed, and these formations appeared like agglomerates of smaller building units. In addition, AFM shows that the cryo-fractured reliefs of PUNFs differed from the reliefs of the pristine PU.

In the photomicrographs, the pristine PU and PUNFs seem to be made up of individual spherical/oval harder conglomerates that we presume are nanoparticles, and these look to be connected by the softer segments. The diameter of these oval formations is, on average, 0.48 μm for PUNF-60-Cu, 0.32 μm for PUNF-60-Zn, 0.92 μm for PUNF-60-CuZn, and 1.13 μm for pristine PU, and their size decreases in the series of the prepared PUNFs. The decrease in size could be connected to the addition of nanoferrites to the PU matrix.

The AFM was used to measure the roughness (average roughness (Ra)) and the root mean square roughness (Rq) of the material surfaces, as shown in Table 7. The surfaces of the PUNFs with added ferrite nanoparticles were smoother than those of pristine PU, indicating the changes in the structure of the NCs caused by this addition. PUNF-60-Zn, which contained the smallest nanoparticles among our materials, was also the least rough of the materials.

### 3.9. Copper, Zinc, and Iron Ion Release

**Figure 7:**
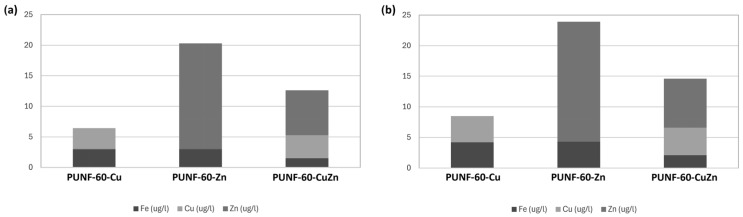
Nanocopper and nanozinc release from PU nanocomposite films (**a**) into deionized water and (**b**) PBS buffer.

The biological effects of nanocopper and nanozinc depend on the release of ions into the surrounding matrix. The release of nanocopper and nanozinc into deionized water and PBS was measured using MP-AES (Figure 7). The evaluation of ion release was performed after 24 h of soaking in deionized water or PBS to ensure that the maximum concentration of ions was released. The results show that the maximum concentration measured did not exceed the toxic dose for humans [22].

Single-element PU/ferrite NC desorption experiments show Fe was released uniformly from all examined NCs, while the release of copper and zinc ions differed between the materials. In addition, the concentration of copper released from PUNFs containing CuFe_2_O_4_ or Cu_0.5_Zn_0.5_Fe_2_O_4_ nanoparticles was similar. The differences or similarities in the concentrations of the released metal ions can be explained in two ways. Firstly, the metal ions used have different interactions with the polymer matrix. Secondly, iron, copper, and zinc have different redox potentials, which influence their reactivity with water molecules and other substances in the solution. These differences affect not only their solubility but also their potential toxicity and bioavailability. The results obtained reflect the synergistic effect of these interactions. The higher amount of zinc released from 1% PUNF-60-Zn compared to other PUNFs makes this material a good candidate for applications where antibacterial properties are paramount. The concentrations of zinc and iron released from 1% PUNF-60-Cu_0.5_Zn_0.5_ were lower compared to the other materials, probably due to the factors already mentioned and the smaller contact availability of both these metals toward molecules of water.

Comparing the desorption in deionized water with that in PBS (Figure 7), more ions were desorbed from the polymers in the buffer than in the water, while the trend of relative concentrations was maintained. This indicates that the concentration of metal ions released in PBS is probably influenced by the greater ionic strength of the buffer in comparison to deionized water. Nevertheless, both desorption experiments suggest that zinc ions display more favorable desorption properties compared to Cu ions, regardless of the solution used for desorption.

### 3.10. Protein Adsorption

**Figure 8:**
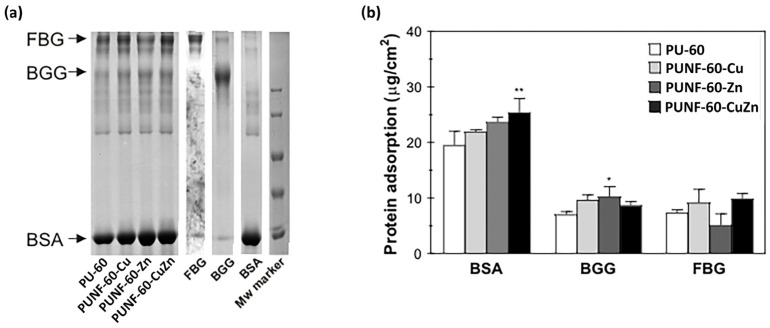
The adsorption of albumin, γ-globulin, and fibrinogen from a three-component protein mixture on pristine PU and PUNF materials: (**a**) electrophoresis of adsorbed proteins and (**b**) competitive adsorption of proteins. * *p* < 0.05 vs. PU-60 sample, ** *p* < 0.01 vs. PU-60 sample.

To assess the impact of copper-, zinc-, and copper–zinc ferrites on the protein adsorption profile of the investigated PU NCs, the materials were incubated in a mixture of three major plasma proteins at physiological concentrations (Figure 8). In general, the addition of ferrite nanoparticles increased the adsorption of most of the investigated proteins, with the exception of FBG in the case of PUNF-60-Zn. All materials preferentially bound BSA, and surface modification with copper-, zinc-, and copper–zinc ferrites increased albumin binding to PUNFs by 13%, 22%, and 30%, respectively. The considerable amount of albumin bound to the materials probably reflects the high concentration of this protein in the protein mixture. Surface passivation with albumin is an important prerequisite for all materials designed for permanent contact with blood, as it indicates thromboresistant properties. The total amount of γ-globulins and fibrinogen bound to the materials was approximately 3 to 4.5 times less than the quantity of adsorbed albumin. In accordance with the pattern of albumin binding, the addition of copper-, zinc-, and copper–zinc ferrites increased the adsorption of γ-globulins by modified PU films by 36%, 45%, and 23%, respectively. Moreover, PU modification with copper- and copper–zinc ferrites increased fibrinogen adsorption by 25% and 36%, respectively. In contrast, the modulation of the PU surface with zinc ferrite decreased fibrinogen binding by 30%, and consequently, the lowest ratio of FBG/BSA binding was associated with PUNF-60-Zn (Table 8). The ability of the blood-contacting material to adsorb more albumin than fibrinogen may indicate the low thrombogenic capacity of the investigated material.

The complex process of protein adsorption on a biomaterial’s surface is influenced by morphology, surface charge, van der Waals interactions, hydrogen bonds, and electrostatic interactions. In this study, the type of embedded nanoparticles had a clear impact on the amount of adsorbed proteins. The results show that PUNFs adsorbed higher amounts of proteins, especially BSA, compared to pure PU. Of the PUNFs examined, the largest amount of BSA was adsorbed by the PUNF-60-CuZn nanocomposite, a sample with the highest WCA and hydrophobicity. As hydrophobic interactions can be an important driving force for protein–biomaterial interactions, the high affinity of PUNF-60-CuZn toward BSA could be correlated with its highest hydrophobicity [58]. Additionally, the results obtained reveal that the hydrophilic surface of the PU/zinc ferrite NCs adsorbed more BSA and the least amount of FBG compared to other PU NCs. Other factors that may influence protein adsorption in addition to hydrophilicity are the morphology and HBI of the prepared PUNF-60-Zn, which changes in the presence of ZnF_2_O_4_ nanoparticles. Hence, the low protein adsorption onto PUNF-60-Zn could possibly reflect the highest HBI of this sample compared to the other PUNFs. The high HBI value of this sample implies a low capacity for hydrogen bonding between the proteins and free urethane, ester CO, and NH groups on the surface. Accordingly, the lower HBI values of PUNF-60-Cu and PUNF-60-CuZn agree with the higher total amount of proteins bound to these samples. In addition, the surface of PUNF-60-Zn was the least rough of all materials analyzed. The smoother surface implies less binding of FBG, as previously observed by Zhang et al. [59], while an *R*_a_ below 50 nm has been suggested by others to be beneficial in preventing platelet adhesion and thrombogenesis [60].

### 3.11. Cytocompatibility

**Figure 9:**
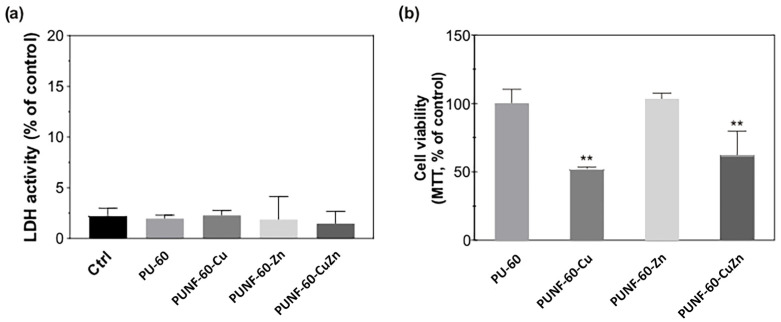
Influence of PU and PUNFs films on (**a**) LDH release and (**b**) mitochondrial activity in EA.hy926 cell line after 48 h. ** *p* < 0.01 vs. PU-60 sample.

The ability of the investigated PU NCs to support the growth and attachment of endothelial cells was assessed using human EA.hy926 endothelial cells. Endothelial cells form the innermost layer of blood vessels, and their growth confers non-thrombogenic properties to the vascular graft. All the prepared films were non-toxic to endothelial cells, as shown by the absence of LDH leakage into the cultivating medium, even after 48 h incubation of the cells with PU materials, and the preserved morphology of the cells growing in close proximity to the materials (Figure S5 in Supplementary Materials). The conventional MTT assay (Figure 9) shows the comparable viability of endothelial cells grown on pristine PU and the PUNF surface modified with zinc ferrite, while the surface modified with copper- and copper–zinc ferrites bound a lower number of cells with active mitochondria than the pristine PU.

**Figure 10:**
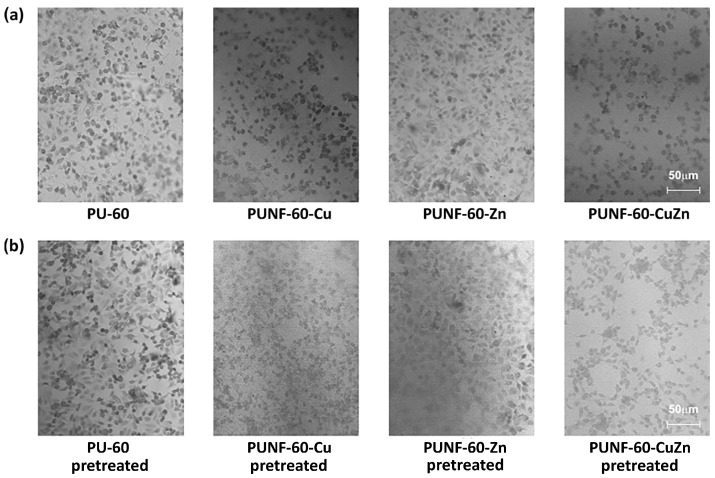
Photographs of pristine PU and PUNF materials with EA.hy926 cells adhered (**a**) on the surface of the materials and (**b**) on the surface of the materials pretreated with a three-component protein mixture.

The observed phenomenon correlates with the results of light microscopy, which show that pristine PU and PUNF-60-Zn support the unrestricted growth of endothelial cells. Indeed, endothelial cells attached to pristine PU and PUNF-60-Zn formed confluent cell monolayers with maintained polygonal morphology. The density of cells adhering to these two films was similar (Table 8). In contrast, the number of cells adhered to PUNF-60-Cu and PUNF-60-CuZn was lower compared to pristine PU and PUNF-60-Zn, as demonstrated in Figure 10a and Table 8. Surface preconditioning with a three-component mixture of blood proteins did not greatly impact on the number of EA.hy926 cells attached to the pure and modified PU NCs, although it slightly improved the attachment of endothelial cells to PUNF-60-Cu and PUNF-60-CuZn. These results indicate that all PU NCs have the potential to unrestrictedly bind endothelial cells in a non-preconditioned state and especially under physiological conditions.

**Figure 11:**
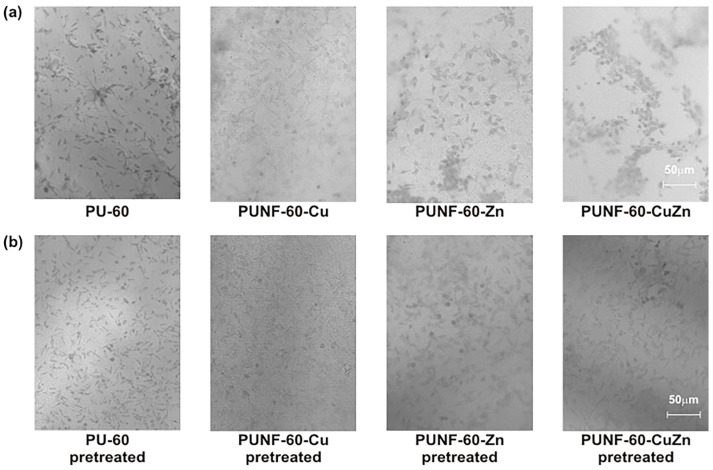
Photographs of PU and PU/ferrite nanocomposite films with fibroblast cells adhered (**a**) on the surface of the materials and (**b**) on the surface of the materials pretreated with three-component protein mixture.

To assess the selectivity of the PUNF materials for different cell types, the films were incubated with the human MRC-5 fibroblast cell line (Figure 11). The optical microscopy results show that pristine PU and PUNF-60-Zn bind the fewest MRC-5 cells under both untreated and pretreated conditions. Copper-ferrite-based PU promoted fibroblast attachment by 116% compared to pristine PU, while preconditioning pristine PU and PUNF-60-CuZn with blood proteins resulted in significantly improved attachment of fibroblasts to their pretreated surfaces.

Changes in the physicochemical properties of materials affect the behavior and fate of cells. For example, stiffness, surface roughness, bulk and surface chemical structures, wettability, hydrophilicity, composition, and degree of microphase separation can alter the behavior and fate of cells [43,61,62]. Adequate surface hydrophilicity and greater microphase separation promote endothelial cell adhesion to biomaterial surfaces [43]. PU-60 and PUNF-60-Zn exhibited the highest DPS among the polymers studied, so the favorable adhesion of endothelial cells may be associated with the lower-phase mixing of these materials. Moreover, the enhanced adherence of EA.hy926 cells to PUNF-60-Zn could be correlated with its moderately hydrophilic surface, a property known to advance cells binding to the surface of biomaterials. In addition, the size of the ferrite nanoparticles incorporated into the PU network could impact the adhesion and growth of endothelial cells by affecting the homogeneity of the investigated materials. The best dispersion of nanoparticles within the NCs was achieved in the case of PUNF-60-Zn. This means that the highest proportion of magnetic nanoparticles was exposed on the surface of PUNF-60-Zn, which, in turn, created a microenvironment with tiny magnetic fields that likely positively affected cell proliferation [63,64]. Finally, stiffer substrates were found to better promote endothelial cell adhesion, proliferation, and spreading compared to less stiff substrates [65], while Tan and Teoh [66] found that fibroblasts prefer to proliferate in a lower stiffness environment that is closer to their natural environment. These results are consistent with those obtained here. Therefore, our study implies that the addition of zinc ferrite nanoparticles imparts adequate microphase separation, increased hydrophilicity of the material, and greater stiffness to the PU matrix, which, in turn, supports the attachment and appropriate proliferation of endothelial cells. Apart from the physicochemical properties, the binding of endothelial cells to the biomaterial in vivo is determined by the proteins adsorbed on the surface. Pretreatment with the mixture of the most abundant blood proteins advanced the binding of endothelial cells to the PUNF-60-Cu and PUNF-60-CuZn samples. This could be a consequence of the FBG binding profile of these nanocomposites. Namely, a larger amount of FBG was adsorbed on the NCs embedded in Cu and CuZn ferrites than on PU-60 and PUNF-60-Zn, and FBG is known to contain arginine–glycine–aspartic acid, a tripeptide that stimulates endothelial cell adhesion and spreading [67].

Taken together, our results imply that, under conditions mimicking physiological, PUNF-60-Zn has the most favorable protein- and cell-binding profile of all the investigated materials and would, therefore, be suitable for vascular implant development.

### 3.12. Antioxidant Activity Results

**Figure 12:**
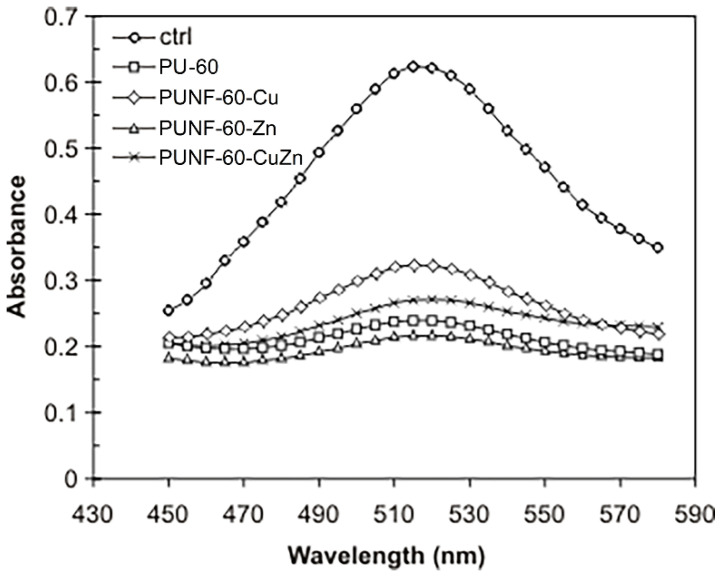
UV–VIS spectra of DPPH solutions in methanol after 30 min incubation in the dark without (ctrl) or with materials.

The implantation of biomaterials in the body may trigger oxidative stress, which leads to inflammation, chronic diseases, and other health problems. In contrast, the free radical scavenging property of biomaterials can improve wound healing and protect tissues from oxidants that can cause inflammation. For this reason, the development and manufacturing of materials with antioxidant properties can play a significant role in improving biocompatibility, while examining the antioxidant activity of biomaterials is of great importance. In this work, the antioxidant activity of the newly prepared PU/ferrite NCs was determined using the DPPH free radical scavenging assay. The assay shows a substantial decrease in the DPPH absorption band intensity in the presence of all investigated materials compared with the control, indicating that pristine PU and PUNF materials efficiently scavenge DPPH radicals (Figure 12). The calculated percentage of DPPH elimination ranged from 61.4% (PUNF-60-Cu) to 76.1% (PUNF-60-Zn) (Table 8).

It is well established that ZnFe_2_O_4_ and CuFe_2_O_4_ nanoparticles exhibit marked antioxidant properties, which emphasizes their potential to attenuate oxidative stress [46,68]. One reason for the detected antioxidant potential of our PUNFs could be their capacity to transfer electrons from the oxygen atom of the nanoparticles to the nitrogen atom of DPPH [46]. Moreover, the ability of antioxidant metal ions to neutralize free radicals has also been demonstrated in previous studies [46,69]. In addition, clear evidence exists for the claim that ZnFe_2_O_4_/cellulose-based nanocomposites are useful natural antioxidants against oxidative stress associated with degenerative diseases [70]. Accordingly, the results obtained show PUNF-60-Zn had the highest antioxidant activity compared to the other prepared PUNFs and pristine PU. Thus, these results suggest that the incorporation of zinc ferrite nanoparticles into the PU matrix significantly improves the antioxidant capacity of PUNFs.

## 4. Conclusions

Different types of spinel ferrite nanoparticles (copper, zinc, and copper–zinc) were successfully incorporated into a thermoset PU matrix that was used to fabricate novel organic–inorganic nanocomposites by an in situ polymerization method.

The presence of ferrite nanoparticles in the PU matrix was confirmed by FTIR analysis. Moreover, the FTIR analysis highlights a strong modification of the poly(siloxane-urethane) morphology in the presence of ferrite nanoparticles. The introduction of ferrite nanoparticles into the PU matrix hinders hydrogen bonding between the urethane carbonyl and -NH groups of the hard segment, causing a decrease in the phase separation of the prepared NCs. The crosslinking density and surface roughness of the PU NCs are reduced compared to pristine PU.

The incorporation of zinc ferrite nanoparticles improves both the nanomechanical and thermal properties of NCs. The following factors confer these improvements: a higher hydrogen bonding index, good nanoparticle dispersion, and a reasonably homogeneous distribution of the zinc ferrite nanoparticles in the PU matrix. The PUNF-60-Zn polymer is harder and stiffer (hardness of 41 MPa and Young’s modulus of 184 MPa) than pristine PU (hardness of 31 MPa and Young’s modulus of 170 MPa), as was proven by nanoindentation studies. The start degradation temperature (T_10%_) of PUNF-60-Zn (T_10%_ = 286 °C) was the highest compared to the other prepared PUNFs (T_10%_ = 283 and 285 °C). Moreover, a reduction in WCA compared to pristine PU and, thus, enhanced hydrophilicity of the surface is associated with the incorporated zinc ferrite nanoparticles of the smallest particle size.

Embedding ferrite nanoparticles into the polymer matrix alters the surface roughness and physicochemical properties of the material, which affects protein adsorption, cell (endothelial and fibroblast) attachment, and antioxidant activity. All the prepared PU NCs preferentially adsorb BSA over the other two blood proteins examined, while their surfaces display excellent resistance to FBG, which is beneficial for hemocompatibility. PU NCs based on zinc ferrite nanoparticles exhibit the most favorable protein adsorption profile and good cytocompatibility. The cellular studies with endothelial cells and fibroblasts reveal the non-toxic chemistry of all the prepared PUNFs, along with the clear potential of these composites to support cell attachment in untreated conditions and after pretreatment with plasma proteins. Among the prepared NCs, the stiffer material has improved cell adhesion. The hydrophilicity and antioxidant activity of zinc ferrite-based PU NCs are both greater compared to pure PU and other prepared NCs, providing a suitable environment for endothelial cell attachment.

The demonstrated non-toxic behavior, good protein binding profile, good cell adhesion, and strong antioxidant activity of the prepared nanocomposites with ferrite nanoparticles, especially with zinc ferrite nanoparticles, show the potential of these NCs to be used as matrices for the preparation of biomedical devices, particularly vascular implants. Since the performance and longevity of vascular implants (vascular grafts and stents) can be improved by coatings with adjustable mechanical strength, smooth surfaces, and demonstrated biocompatibility [71,72], our study strongly proposes that PUNFs, especially PUNF-60-Zn, may be suitable materials for this application. Further steps in their evaluation should include preclinical testing of their biological safety and performance using in vitro and in vivo approaches [73]. Polymer coating of the scaffold of interest (graft or stent) by electrospinning or dipping techniques could be exploited to obtain material in a form similar to the final shape for further evaluation in animal models. In summary, by varying the surface and/or PU composition as well as the ferrite nanoparticle content, the prepared NCs gained adequate stiffness, hardness, smoothness, relative hydrophilicity, and biocompatibility. All these modifications would presumably minimize friction and improve the long-term performance of the implant when applied in the form of a coating. Therefore, PUNFs should be further investigated to develop them as a biocompatible coating platform for various biomedical applications.

## Figures and Tables

**Table 1:** Band assignation in the FTIR spectra of PUNFs.

Band Width, cm^−1^	Band Assignation
3324	stretching vibrations of hydrogen-bonded urethane N-H groups
3450	stretching vibrations of non-hydrogen urethane N-H groups
2960	asymmetric stretching vibrations of CH_2_ groups
2865	symmetric stretching vibrations of CH_2_ groups
1735	stretching vibrations of free C=O groups of urethane bonds
1725	stretching vibrations of free C=O groups of ester bonds
1715	stretching vibrations of hydrogen-bonded urethane C=O groups in disordered hard domains
1700	stretching vibrations of hydrogen-bonded C=O groups of urethane bonds in ordered hard domains
1650	stretching vibrations of hydrogen bonded C=O groups of ester bonds
1590	C=C stretching vibrations in the aromatic rings
1530	amide II vibrations
1415	C=C stretching vibration in the aromatic rings
1230	amide III vibrations
1105	stretching vibrations of C-O-C and Si-O-Si bands
1010	stretching vibrations of C-O-C and Si-O-Si bands
800	rocking vibration of C-H in SiCH_3_
534	vibration of Fe-O band
580	stretching vibration of the M-O band in the octahedral sites; (M=Zn, Cu)
490	stretching vibration of the M-O band in the tetrahedral sites; (M=Zn, Cu)

**Table 2:** Deconvolution results for the C=O and -NH stretching regions of the FTIR spectra of prepared materials.

Material	CO_free urethane_ (1735 cm^−1^)	CO_free ester_ (1725 cm^−1^)	CO_order urethane_ H-Bonding (1700 cm^−1^)	CO_disorder urethane_ (1715 cm^−1^)	CO_ester H-bonding_ (1650 cm^−1^)	NH_H-bonding_ (3315 cm^−1^)	NH_free_ (3440 cm^−1^)	HBI ^a^ (%)	DPS ^b^ (%)
PUNF-60-Cu	39.4	14.1	22.8	16.2	7.8	91.1	8.9	40.8	49.7
PUNF-60-Zn	31.2	13.4	34.8	12.5	8.1	94.0	6.0	80.0	60.3
PUNF-60-CuZn	34.2	14.8	23.8	15.8	9.4	85.8	14.2	47.6	53.7
PU-60	30.2	13.9	35.9	13.2	6.8	95.1	4.9	82.7	61.9

^a^ HBI, i.e., hydrogen bonding index of urethane groups; HBI = COorder urethane H-bonding/(COfree urethane + COdisorder urethane). ^b^ DPS, i.e., degree of phase separation; DPS = ACO(1700) + ACO(1715)/(ACO (1700) + ACO(1715) + ACO(1735).

**Table 3:** Model independent structure parameters obtained from the small-angle part of the SWAXS measurements.

Sample	Rg [1/nm]	I0^+^ [arb. Units]	k1	k2	D [nm]	R_1m_ [nm]	Rg * [1/nm]	I0 * [arb. Units]	k1 *	k2 *	D * [nm]	R_1m_ * [nm]
PU-60	28	4.4	−3.1		10	28						
PUNF-60-Cu	28	88	−2.6	−3.7	10	11	28	98	−2.6	−3.7	8	9
PUNF-60-Zn	30	64	−3.3		9	19	31	82	−3.3		9	11
PUNF-60-CuZn	31	50	−3.6		10	16	34	75	−3.7		10	13

* Parameters obtained from PU/ferrite nanocomposites after subtracting the scattering caused by pristine PU (see the text for further explanation).

**Table 4:** Nanomechanical properties of pristine PU and PUNF films.

Sample	Young’s Modulus, MPa	Hardness, MPa	Plasticity (Ductility Index), MPa
PUNF-60-Cu	152	31	4.90
PUNF-60-Zn	184	41	4.49
PUNF-60-CuZn	142	27	5.25
PU-60	170	31	5.48

**Table 5:** Summarized results of TGA, DTG, and DSC for the prepared PUNFs and pristine PU.

Sample	T_10%_, °C	T_50%_, °C	T_90%_, °C	T_max_, °C	Residual Weight, 650 °C wt.%	T_gHS_ (DSC), °C
PUNF-60-Cu	283	365	480	289/310/375/444	6.48	29
PUNF-60-Zn	286	368	480	290/305/389/441	6.11	27
PUNF-60-CuZn	285	368	486	305/324/389/430	5.84	26
PU-60	287	385	459	260/306/361/431	4.33	29

**Table 6:** Swelling degree, density, ρPUNF, values of VPUNF, ν, and Mc calculated from swelling measurements of the prepared PUNFs and pristine PU.

Sample	Swelling Degree	*ρ*, g/cm^3^	*V*_PUNF_	*ν* × 10^3^, mol/cm^3^	*M*_c_, g/mol	Water Absorption, wt.%
PUNF-60-Cu	7.0895	1.1702	0.0674	4.55	257.37	4.11
PUNF-60-Zn	7.8046	1.0437	0.0594	4.14	252.20	5.99
PUNF-60-CuZn	2.6949	1.1220	0.1621	9.17	122.30	3.20
PU-60	2.8762	0.9209	0.1913	10.60	86.88	2.21

**Table 7:** Water contact angle (WCA) and surface roughness measurements made by AFM for the PUNF materials and pristine PU.

Material	WCA (°)	Ra (nm)	Rq (nm)	Avg. Height (nm)	Peak to Peak (nm)
PUNF-60-Cu	91	8.77	11.89	69.94	155.43
PUNF-60-Zn	86	6.33	9.72	37.79	110.61
PUNF-60-CuZn	94	82.26	108.5	294.9	756.3
PU-60	97	137.72	165.2	445.5	895.5

**Table 8:** FBG/BSA ratio, cell density, and the percent of DPPH scavenging of the prepared materials.

Material	FBG/BSA Ratio	Cell Density (EA.hy926 Cells) (cells/mm^2^) Untreated	Cell Density (EA.hy926 Cells) (cells/mm^2^) Pretreated	Cell Density (MRC-5 Cells, Fibroblast Cell Line) (cells/mm^2^) Untreated	Cell Density (MRC-5 Cells, Fibroblast Cell Line) (cells/mm^2^) Pretreated	DPPH Scavenging (%)
PU-60	0.373	3250.0 ± 208.2	3356.3 ± 115.5	1783.3 ± 386.9	2700 ± 216.8	68.2 ± 0.7
PUNF-60-Cu	0.416	2318.1 ± 282.8	2871.5 ± 230.2	3866.7 ± 647.2	3766.7 ± 454.6	61.4 ± 1.6
PUNF-60-Zn	0.217	3195.2 ± 228.0	3272.2 ± 240.8	2050.0 ± 620.9	2783.3 ± 296.6	76.1 ± 0.6
PUNF-60-CuZn	0.390	1848.2 ± 270.2	2323.3 ± 355.9	2216.7 ± 951.2	3983.3 ± 503.0	63.8 ± 2.0

## Data Availability

The original contributions presented in this study are included in the article/Supplementary Materials. Further inquiries can be directed to the corresponding author(s).

## Supplementary Materials

The following supporting information can be downloaded at: https://www.mdpi.com/article/10.3390/polym17020152/s1, Figure S1: TEM micrograph of CuFe_2_O_4_, ZnFe_2_O_4_, and Cu_0.5_Zn_0.5_Fe_2_O_4_ nanoparticles; Figure S2: X-ray diffraction pattern of CuFe_2_O_4_, ZnFe_2_O_4_, and Cu_0.5_Zn_0.5_Fe_2_O_4_ nanoparticles; Figure S3: X-Ray scattering of the prepared PU/ferrite nanocomposites and pure PU films in the whole small-angle and near wide-angle experimental region plotted as log(I) vs. 2θ as is commonly used in XRD; Figure S4: Small-angle region of the SWAXS measurements of the prepared PU/ferrite nanocomposites after subtraction of the pure PU films scattering; Figure S5: Images of pure PU and PU/ferrite nanocomposite films with EA.hy926 cells adhered around the PU and PU/ferrite nanocomposite films.
