# Supplementary material for: Structure and Functional Characteristics of Novel Polyurethane/Ferrite Nanocomposites with Antioxidant Properties and Improved Biocompatibility for Vascular Graft Development
Source: Polymers (Basel). 2025 Jan 9;17(2):152. doi: 10.3390/polym17020152 (PMC11768855; doi:10.3390/polym17020152)
Supplement: Supplementary file 1 [file polymers-17-00152-s001.zip › polymers-3404908-supplementary.pdf]

Structure and Functional Characteristics of Novel Polyurethane/Ferrite Nanocomposites with Antioxidant Properties and Improved Biocompatibility for Vascular Graft Development

Marija V. Pergal [1,*], Jelena Brkljačić [2], Dana Vasiljević-Radović [1], Miloš Steinhart [3], Sanja Ostojić [4], Biljana Dojčinović [1], Bratislav Antić [5] and Gordana Tovilović-Kovačević [2,*]

[1] Center for Microelectronic Technologies, Institute of Chemistry, Technology and Metallurgy—National Institute of the Republic of Serbia, University of Belgrade, Njegoševa 12, 11000 Belgrade, Serbia; dana.vasiljevic@ihtm.bg.ac.rs (D.V.-R.); bmatic@chem.bg.ac.rs (B.D.)

[2] Department of Biochemistry, Institute for Biological Research "Siniša Stanković"—National Institute of the Republic of Serbia, University of Belgrade, Bulevar despota Stefana 142, 11060 Belgrade, Serbia; brkljacic@ibiss.bg.ac.rs

[3] Institute of Macromolecular Chemistry CAS (IMC), Heyrovsky Sq. 2, 16206 Prague 6, Czech Republic; stein@buphy.bu.edu

[4] Institute of General and Physical Chemistry, University of Belgrade, Studentski trg 12-16, 11000 Belgrade, Serbia; ostojicsanja404@gmail.com

[5] The VINČA Institute of Nuclear Sciences—National Institute of the Republic of Serbia, University of Belgrade, Mike Petrovića Alasa 12-14, 11001 Belgrade, Serbia; bantic@vin.bg.ac.rs

* Correspondence: marijav@chem.bg.ac.rs or marija.pergal@ihtm.bg.ac.rs (M.V.P.); tovilovicg@ibiss.bg.ac.rs (G.T.-K.)

Supplementary material

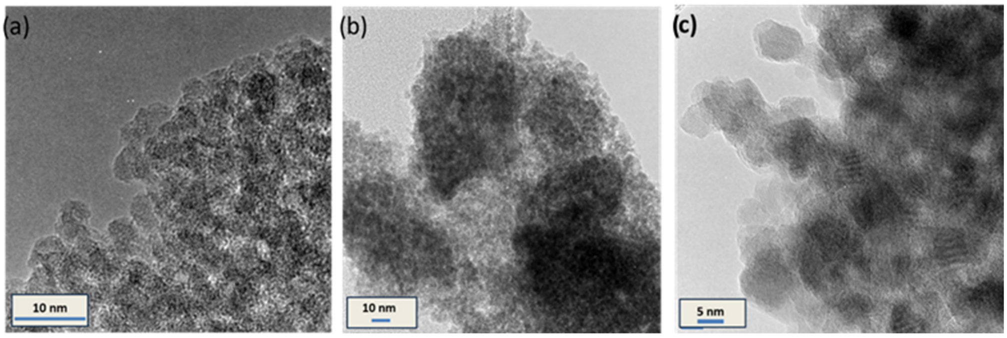

Figure S1. TEM micrograph of: a) $CuFe_2O_4$, b) $ZnFe_2O_4$ and c) $Cu_{0.5}Zn_{0.5}Fe_2O_4$ nanoparticles.

Particle size and shape were studied by transmission electron microscopy (TEM) using a 200 keV JEOL-2000 FXII microscope. TEM samples were prepared by placing one drop of a dilute suspension of nanopowder in acetone, previously sonicated for 30 minutes, on a carbon coated copper grid. The solvent was allowed to evaporate slowly at room temperature.

X-ray powder diffraction data (XRPD) were collected using a high-resolution Smart Lab® diffractometer (Rigaku, Japan), equipped with Cu Kα radiation source (λ = 1.5406 Å) under a voltage of 40 kV and a 30-mA current. The data collection for the patterns was performed in the 10-70° 2θ range.

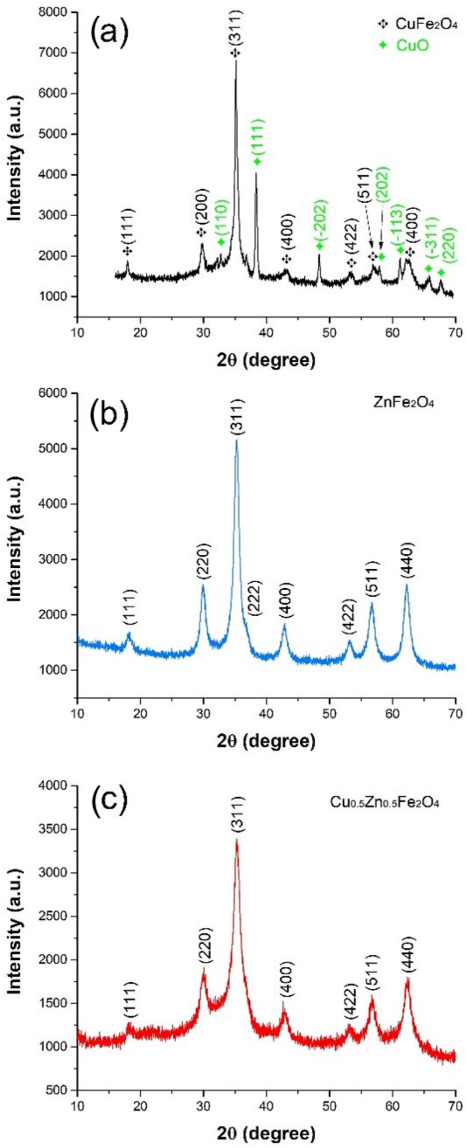

Figure S2. X-ray diffraction pattern of CuFe2O4, ZnFe2O4 and Cu0.5Zn0.5Fe2O4 nanoparticles.

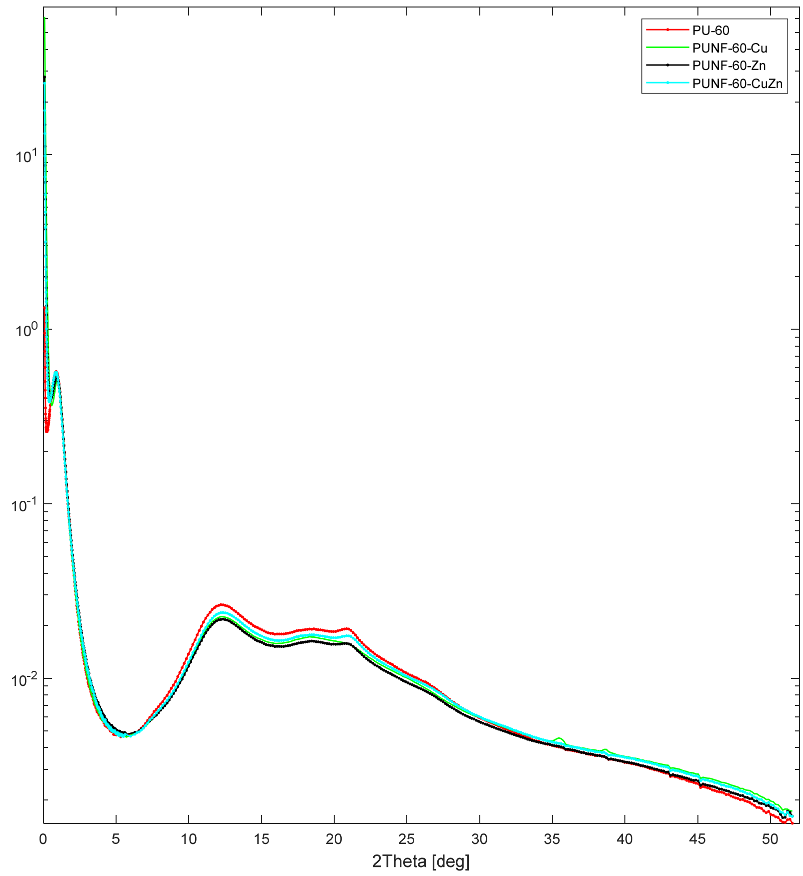

Figure S3. X-Ray scattering of the prepared PU/ferrite nanocomposites and pure PU films in the whole small-angle and near wide-angle experimental region plotted as log(I) vs 2 as is commonly used in XRD and also in Figure S2.

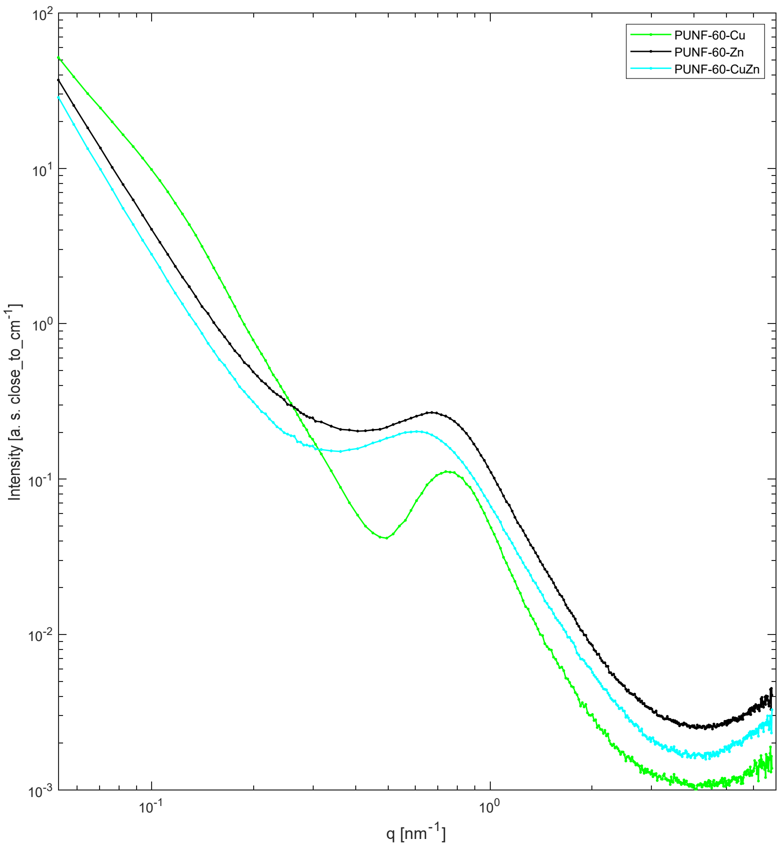

Figure S4. Small-angle region of the SWAXS measurements of the prepared PU/ferrite nanocomposites after subtraction of the pure PU films scattering.

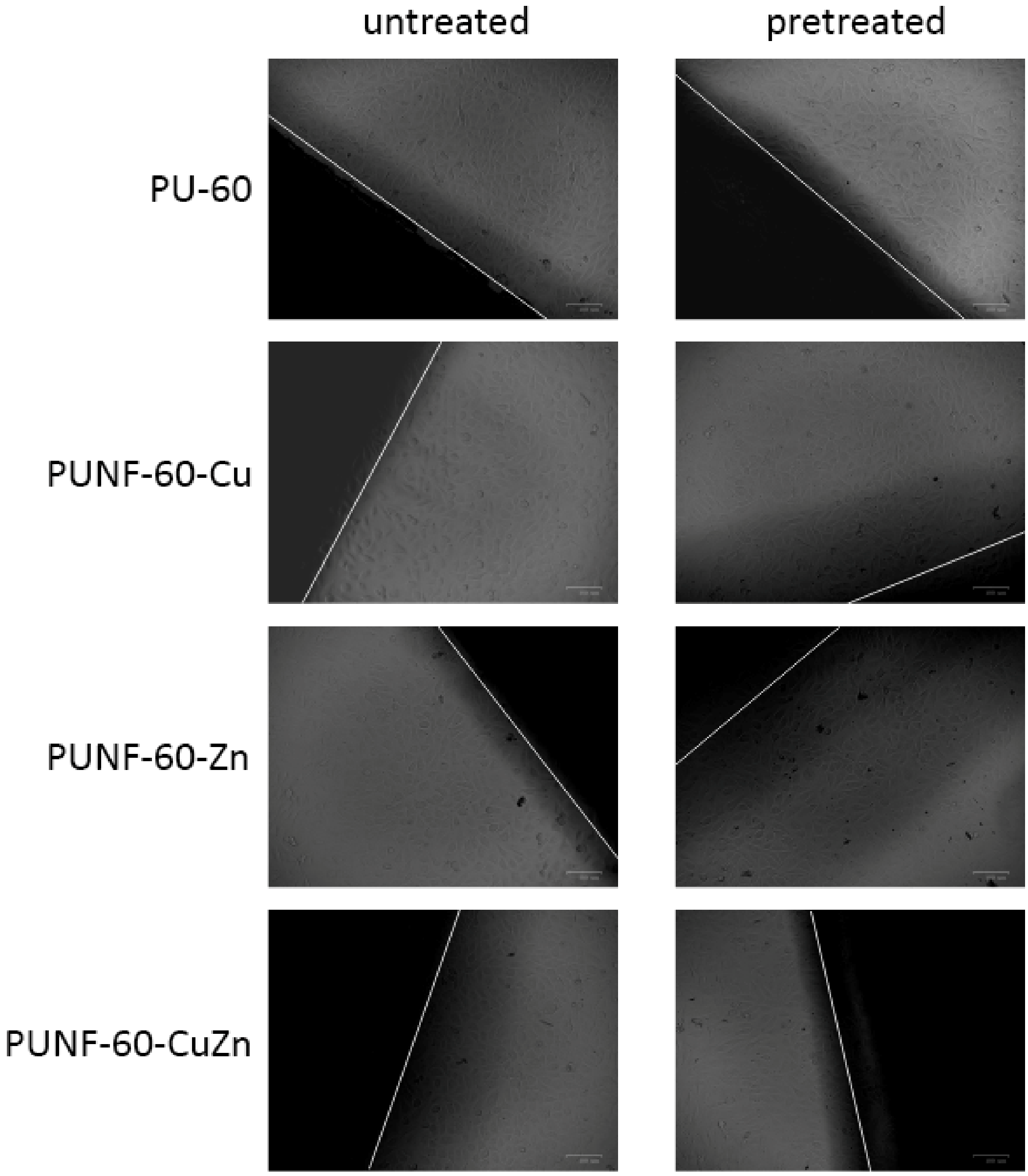

Figure S5. Photographs of pure PU and PU/ferrite nanocomposite films with EA.hy926 cells adhered around the of PU and PU/ferrite nanocomposite films.
